# How Electronic Cigarette Affects the Vascular System

**DOI:** 10.1155/2022/3216580

**Published:** 2022-09-30

**Authors:** Vito Anggarino Damay, Setiawan Setiawan, Ronny Lesmana, Mohammad Rizki Akbar, Antonia Anna Lukito

**Affiliations:** ^1^Department of Cardiovascular Medicine, Pelita Harapan University, Banten, Indonesia; ^2^Department of Biomedical Sciences, Padjajaran University, Bandung, Indonesia; ^3^Department of Cardiology and Vascular, Padjajaran University, Bandung, Indonesia

## Abstract

The popularity of the electronic cigarette has soared in the last decades. However, the health effect of smoking electronic cigarettes on the vascular system is unclear. This systematic review examines the electronic cigarettes' effect on the vascular system from recent evidence. A systematic search was conducted in MEDLINE (PubMed) database from January 2016 to August 2021 for studies assessing the vascular effect of chronic use of electronic cigarettes on human and animal. The Cochrane Risk of Bias 2, NIH Quality Assessment for Cross-Sectional Study, and SYRCLE's Risk of Bias were used to assess the risk of bias in interventional, observational, and animal study, respectively. A narrative synthesis of evidence is provided to describe results. From 101 retrieved studies related to electronic cigarettes effect on the vascular system, a total of 16 studies are included in this review. The overall results indicated that electronic cigarette use is associated with adverse events in the vascular, including the incident of elevated oxidative stress, endothelial dysfunction, inflammation, arterial stiffness, and the development of atherosclerotic lesion. Further studies should broaden perspectives and reveal more about the mechanism of how electronic cigarettes impact on vascular system.

## 1. Introduction

Cardiovascular diseases (CVDs) are a leading cause of mortality globally and responsible for up to 32% of all deaths in the world [1]. One of the major risk factors for CVDs is smoking tobacco. In particular, smoking is associated with the development of atherosclerosis and is known to induce oxidative stress and damage endothelium, which can be observed clinically [2]. Preventive action is necessary to reduce the risk of CVD, mainly by smoking cessation. Smoking cessation is effective in lowering the risk of CVD among smokers when done earlier than 5 years [3]

In recent decades, the electronic cigarette has gained popularity as it is marketed as an alternative to tobacco smoking. The electronic cigarette is a battery-powered device that heats refillable premix liquid (e-liquid) to aerosol. Major compounds in e-liquid include propylene glycol, ethylene glycol, glycerol, flavors, and optional nicotine in various concentrations [4]. The aerosol produced is inhaled by users and the action is commonly known as ‘vaping'.

Since being introduced to the market in the mid-2000s, many people from all over the world have gained access to the device. Users may range from as young as youths to young adults and adults. In the United States and European countries, the electronic cigarette use is more prevalent among males, younger adults, current smokers, and former smokers [5, 6]. In a survey by Etter and Bullen [7], 96% of 3587 users considered the electronic cigarette as a means of smoking reduction or smoking cessation.

Considering the adverse effects of smoking in general, the health effect of using electronic cigarettes is likewise questionable. Furthermore, the e-liquid, aerosol, and smoke of electronic cigarettes of various brands have been found to contain potentially damaging compounds, such as tobacco-specified nitrosamines (TSNAs), aldehyde, metals, volatile organic compounds (VOCs), and tobacco alkaloids in variable amounts [8].

The electronic cigarette is deemed a safer option than conventional cigarettes, though not completely safe [9]. However, the effect of the electronic cigarette use on the vascular system has remained unclear. There is a potentially worrying effect of electronic cigarettes, yet the evidence is still limited [10]. In this article, we performed a systematic review to explore recent literature on the effect of the electronic cigarette, specifically on the vascular system.

## 2. Methods

### 2.1. Study Selection

Relevant literature published from January 2016 to August 2021 was obtained from the MEDLINE (PubMed) database. Keywords used on search strategy are (“Electronic Nicotine Delivery Systems”[Mesh] OR “Vaping”[Mesh] OR “E-Cigarette Vapor”[Mesh] OR e-cigarette∗[tw] OR “electronic cigarette∗”[tw] OR “e-cigarette vapor”[tw] OR vape[tw] OR vapes[tw] OR e-cigs[tw] OR mods[tw] OR “vape pens”[tw] OR “tank systems”[tw] OR “electronic nicotine delivery systems”[tw]) AND (“Blood Vessels”[Mesh] OR “Arteries”[Mesh] OR “Vascular Stiffness”[Mesh] OR “Atherosclerosis”[Mesh] OR aorta[tw] OR “vascular effect∗”[tw] OR “endothelial function”[tw] OR “arterial stiffness”[tw] OR “coronary artery”[tw] OR “smooth muscle cell” [tw]). The studies gathered from the database search were exported to the Mendeley reference manager and screened for duplicates. Articles are manually selected that contain relevant studies related to the electronic cigarette and its impact on the vascular system.

Initial screening was done based on article type, title, and abstracts, followed by full-text screening. Studies on cells, case series, case reports, and irrelevant studies were excluded. Included articles must evaluate the chronic impact of electronic cigarettes on the vascular system in terms of clinical adverse events among adult or animal populations within 2016 and 2021. The data was input manually by author.

### 2.2. Risk of Bias Assessment

Studies are differentiated into human randomized interventional study (randomized controlled trial or RCT), human observational cross-sectional study, and animal interventional study. The Cochrane Risk of Bias 2 (RoB 2) was used for human RCTs, NIH Quality Assessment for Cross-Sectional Study for human observational cross-sectional study, and SYRCLE's Risk of Bias (SYRCLE's RoB) for animal interventional study.

### 2.3. Data Extraction

Data were extracted from included articles with a predetermined standardized data form. Extracted data include author, year of publication, characteristics of participants, number of participants, type of interventions, nicotine concentration, length of exposure, and main conclusion. Studies are grouped into human studies or animal studies. Results were described narratively because of the nature of the included studies.

### 2.4. Systematic Search

**Figure 1:**
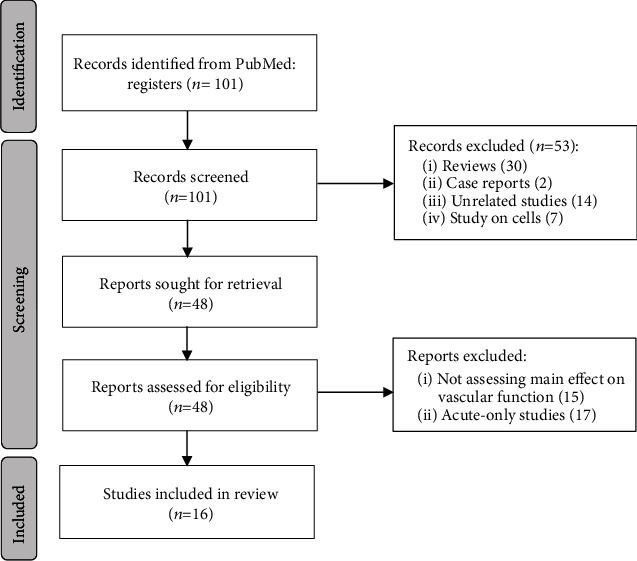
PRISMA Flow Diagram of study selection.

The initial search showed 101 results in PubMed from January 2016 to October 2021. A total of 101 articles were selected further to be screened by content. After the full-text screening, 16 articles were included in this systematic review. The PRISMA Flow Diagram is provided in Figure 1.

### 2.5. Literature Characteristics

The characteristics of the articles included are available in Table 1. There are interventional and observational studies. Interventional studies recruited occasional to heavy smokers and used model mice when assessing the chronic effects of electronic cigarettes with and/or without nicotine. Observational studies recruited nonsmokers and chronic smokers to observe chronic effects of electronic cigarettes.

In interventional human studies, studies lasted one to four months with the nicotine level used in electronic cigarettes varying in concentration from 0.12-16 mg/mL. Meanwhile, in interventional animal studies, the period of studies was between 5 days to 60 weeks and the nicotine concentration ranged from 6 to 24 mg/mL and 2.4% to 4%. All studies measured various clinical parameters and biomarkers on the vascular function, mostly including arterial stiffness, endothelial dysfunction, inflammation, and atherosclerosis.

## 3. Results

In human studies, the type of studies can be interventional (randomized controlled trials or RCTs) and observational. The interventional studies evaluating vascular effects of electronic cigarettes essentially used various designs, which caused differences in the number of participants, type of electronic cigarettes used, nicotine concentration in electronic cigarettes, length of study, and employed vascular markers. The observational studies also varied in the number and characteristics of participants, e.g. smokers and nonsmokers.

Animal studies using mice models that evaluated the vascular-related effect of electronic and conventional cigarettes are all interventional. Similar to human interventional studies, the design used in the studies varied in the number of mice, the composition of electronic cigarette vapor, nicotine concentration, length of study, and observed vascular markers.

### 3.1. Effects on Vascular System

In human studies, despite the difference in the number of participants, nicotine concentration in electronic cigarettes, and length of study, all three RCT studies concluded that electronic cigarettes caused less development in arterial stiffness and endothelial dysfunction compared to conventional cigarettes [11–13]. In regards to the nicotine effect, only one study found that electronic cigarettes without nicotine had no better impacts on health than electronic cigarettes with nicotine [11], while other studies did not focus on the effect of nicotine.

Four latest observational prospective studies provided level IIB evidences of adverse vascular effects related to electronic cigarette smokers compared to nonsmokers, including increased arterial stiffness (*p* = 0.003), development of carotid plaque (*p* < 0.0001), microvascular endothelial dysfunction, and reduced endothelial nitric oxide (eNOS) levels (2.6%; *p* = 0.018) that related with endothelial dysfunction [15, 17–19]. One study did not compare effects of using electronic cigarettes with nonusers but observed that the markers of platelet activation (29%; *p* = 0.04), oxidative stress (23%; *p* = 0.02), and endothelial dysfunction (16%; *p* = 0.02) of electronic cigarette users were lower than conventional cigarette users [16]. The study by Rader et al. [18] became the only one reporting a more significant coronary microvascular endothelial dysfunction in chronic electronic cigarette smokers. Thus, the research demonstrated that electronic cigarette smoking leads to vascular damage significantly, compared with nonsmokers.

All interventional animal studies have differences in study design, particularly nicotine concentration and duration of the study. There is a consistent conclusion that there were unfavorable effects of electronic cigarette use [21–26]. Observed adverse vascular outcomes as summed from those studies, including damaged endothelium-dependent and endothelium-independent vasodilation [21], increased oxidative stress [21–23], increased inflammation [22, 24], increased endothelial dysfunction [22, 23], development of atherosclerotic lesions [22, 24], and increased arterial stiffness [25].

The study by Kuntic et al. [23] is also worthy to note as it is the only study that reported increased oxidative stress by NOX-2 mechanism within 5-days observation, the shortest period among other chronic animal studies. In contrast, one study specifically noted that the effect of electronic cigarettes on some vascular health markers, which included arterial stiffness, inflammation, and oxidative stress, was small to absent [26]. Regarding the nicotine effect, two studies reported that more significant disadvantageous outcomes were observed in electronic cigarettes with nicotine than without nicotine [21, 22].

### 3.2. Other Adverse Events

There are other unfavorable health effects of electronic cigarette use indicated by observational human studies, including association with stroke [14] and myocardial infarction [20]. Another adverse outcome from animals studies was observed by one study [21] in which electronic cigarette vapor caused the development of cardiac hypertrophy.

### 3.3. Risk of Bias Assessment

Risk of bias assessment was done according to the type of the included studies. Based on the assessment using RoB 2, two studies [11, 12] have some concerns for bias and one study [13] was judged as high risk for bias. The summary of this result is available in Table 2.

For observational study, the NIH Quality Assessment was used to determine the quality of studies.  Table 3 shows the summary of the quality assessment. Two studies were judged as good [15, 16], three studies were fair [14, 17, 19], while two studies were deemed as poor [18, 20].

Animal studies were assessed with SYRCLE's RoB as summarized in Table 4. Most studies have unclear to high risk of bias results, especially on items about randomization, allocation concealment, and blinding protocol.

## 4. Discussion

This systematic review explored the vascular effect of the use of electronic cigarettes on humans and animals (mice). Overall, there are potential adverse effects on arterial stiffness and endothelial function from the use of electronic cigarettes [15, 17–19, 21–26], though a few studies noticed its more pronounced effect was attributable to the presence of nicotine [21, 22]. In addition, studies that compared the vascular effect of electronic cigarettes to conventional cigarettes showed consistent results in favor of electronic cigarettes [11–13, 16, 19, 20]. However, it does not provide clear evidence on whether electronic cigarette smoking is harmless to the vascular system.

Contact with combustion products of conventional cigarette is one of the primary sources of its harm [27]. Likewise, the unfavorable impact of e-cigarettes on vascular health found in both human and animal studies may possibly be related to the products of e-liquid heating. The heating of propylene glycol (PG) and glycerol (Gly) produces short-lived free radicals and concerning compounds including formaldehyde, acetaldehyde, and acrolein among other compounds that are detrimental when inhaled at certain concentrations [28–30]. Formaldehyde, acetaldehyde, and acrolein can generate oxidative stress and form adducts with protein, RNA, DNA, which impair cell function [31, 32]. In some e-cigarettes, traces of tobacco-specific nitrosamines (TSNAs), volatile organic compounds, and metals are also found in their aerosol or vapor, which may exhibit toxicity [8, 33, 34].

It is generally known that the electronic cigarette does not produce carbon monoxide (CO) as there is no tobacco combustion. However, recent studies showed otherwise by reporting the presence of potential carbon monoxide from electronic cigarettes. Son et al. [35] also showed that several electronic cigarette brands emitted carbon monoxide, alongside carbonyls, in varying amounts between 0 to 30 *μ*g/puff in a normal vaping condition. In the study by Casebolt et al. [36] the CO concentration can reach over 180 ppm after e-liquid is heated. Carbon monoxide can attach strongly to hemoglobin in the place of oxygen, which can result in reduced oxygen availability in blood. So far, there have been known cases of carbon monoxide reaching a toxic level that may lead to cardiovascular complications, including functional anemia, angina pectoris, congestive heart failure, increased ventricular ectopy, and reduced ventricular fibrillation threshold [27].

Nonetheless, exposure to harmful substances generated from heated e-liquid is known to be lower than from combusted tobacco leaf [37]. Combusted tobacco leaf in conventional cigarettes generates more than 7,000 chemicals, which can still be highly varied depending on initial compounds inside the tobacco blends and whole cigarette components [27, 38]. The difference in generated products of e-liquid and tobacco leaf is evidence for smokers to switch from conventional cigarettes to electronic cigarettes [11–13, 16, 19, 20]. However, long-term exposure to the mentioned e-liquid heating byproducts, though in little amounts, still explains the health chronic effects that are supported by the rest of the studies [15, 17–19, 21–26].

Another explanation of the adverse effects of e-cigarettes possibly lie in the presence of nicotine. A study investigated nicotine exposure on smokers and found nicotine exposure by spray and smoking can induce short-term endothelial dysfunction [39]. In obese rats, nicotine administration caused a further rise in oxidative stress, inflammation, and endothelial dysfunction markers, probably by a pathway involving TNF-*α* [40]. Interestingly, two animal studies also provided histological results showing the development of atherosclerotic lesions from electronic cigarette with nicotine use [22, 24]. Using Oil Red O with hematoxylin and fast green staining, Espinoza-Derout et al. [22] found notable development of atherosclerotic lesion area in the aortic root of mice exposed to 2.4% nicotine electronic cigarette vapor in contrast to saline treatment. Li et al. produced similar results using the same concentration of electronic cigarette vapor, showing increased atherosclerotic lesions in the whole aorta by Oil Red O staining and inside the aortic root by hematoxylin and eosin staining in mice in contrast to air-exposed mice. The effect of nicotine is also associated with atherosclerosis by causing inflammation.

Nicotine is known to induce the generation of inflammation-related factors, such as C-reactive proteins, which have a role in the development of atherosclerosis. An animal study by Catanzaro et al. supported that nicotine has contributed to the development of atherosclerosis as evident by the buildout of aortic lesions. Wu et al. concluded that nicotine stimulates ROS production and NLRP3 activation to possibly incite cellular pyroptosis after conducting an experiment on ApoE^−/−^ mice and human aortic endothelial cells. Besides atherosclerosis, nicotine is thought to induce smooth muscle cells to proliferate by modulating angiotensin II. Angiotensin II is able to activate complex pathways, including Nox5 activation, ROS production, and inflammatory proteins, that eventually cause a rise in oxidative stress and endothelial dysfunction. These possible mechanisms may explain the more notable adverse outcomes observed in a few animal studies. Evidences gathered in this review indicate that chronic use of electronic cigarettes may lead to unfavorable effects on the vascular, especially elevation of oxidative stress and inflammation that impact vascular damage. So far, evidences on adverse events mostly come from nonrandomized observational and animal studies. Although there is a suggestion that electronic cigarettes are less harmful than conventional cigarettes, smoking an electronic cigarette still induces unbeneficial effects to the vascular system. Our study is in line with previous study about impact of electronic cigarettes on vascular damage as a risk factor of cardiovascular disease. Even though using an electronic cigarette is apparently safer than conventional cigarettes, previous research demonstrated that the molecular changes on the cardiovascular system clearly leads to oxidative stress and inflammation [41]. More studies about the impact of electronic cigarette smoking on the vascular system will describe the mechanism with more details.

The restriction of including only studies in the last five years was made to focus on updates from recent evidence regarding electronic cigarette effects on the vascular, nonetheless, a few studies also mentioned cardiac effects. Notwithstanding, this review still has limitations. The outcomes of included studies were highly diverse, thus making them unfit for meta-analysis. A descriptive review has been provided instead to summarize current updates on this topic.

## 5. Conclusion

Evidences from several RCTs seemingly support the popular belief that electronic cigarettes have less effect on the vascular system when compared to conventional cigarettes. However, exposure to electronic cigarettes can cause adverse effects, such as elevated oxidative stress, endothelial dysfunction, inflammation, arterial stiffness, and the development of atherosclerotic lesion. Future studies should broaden perspectives and reveal more about the mechanism of how electronic cigarettes impact the vascular system.

## Figures and Tables

**Table 1:** Characteristics of included study.

#	Reference	Sample		Control group	Nicotine	Length of exposure	Overall risk of bias/quality∗	Result
		Characteristics	Total number of electronic cigarette population					
Human studies								
Randomized controlled trial (RCT)								
1	George et al. [11]	Smokers	74	N/A	16 mg/mL	Chronic (1 month)	Some concerns	Switching from conventional cigarette to electronic cigarette improves endothelial function and vascular stiffness marker.
2	Ikonomidis et al. [12]	Smokers	40	N/A	12 mg/mL	Chronic (4 months)	Some concerns	Switching from conventional cigarette to electronic cigarette improves arterial stiffness and oxidative stress.
3	Ikonomidis et al. [13]	Smokers	70	Tobacco smokers	0.12 mg/mL	Chronic (1 month)	High risk	Replacing conventional cigarette with electronic cigarette results in reduced systolic and oxidative stress.
Observational; cross-sectional								
4	Bricknell et al. [14]	Nonsmokers and smokers	74,013	N/A	N/A	Observational study	Fair	ENDS (including electronic cigarette) use is associated with stroke.
5	Fetterman et al. [15]	Nonsmokers and smokers	36	N/A	N/A	Observational study	Good	Electronic cigarette use is associated with elevated augmentation index (AIx, arterial stiffness marker) and an indication of endothelial dysfunction.
6	Oliveri et al. [16]	Smokers	144	Tobacco smokers	N/A	Observational study	Good	Electronic cigarette users show lower levels of biomarkers of exposure (NNK, nicotine, acrolein, and carbon monoxide) and biomarkers of potential harm (platelet activation, oxidative stress, and endothelial function) than cigarette smokers
7	Podzolkov et al. [17]	Nonsmokers and smokers	2	N/A	N/A	Observational study	Fair	Smoking traditional and electronic cigarette are related to albuminuria and an increase in the augmentation index (arterial stiffness marker).
8	Rader et al. [18]	Nonsmokers and smokers	35	N/A	N/A	Observational study	Poor	Electronic cigarette users show more pronounced impaired coronary microvascular endothelial function than chronic conventional cigarette users.
9	Sahota et al. [19]	Nonsmokers and smokers	20	N/A	4-36 mg/mL	Observational study	Fair	Electronic cigarette users develop more carotid plaque burden than nonsmokers
10	Vindhyal et al. [20]	Nonsmokers and smokers	401 and 2,240 dual users	N/A	N/A	Observational study	Poor	Electronic cigarette users are associated with an increased risk of myocardial infarction than nonsmokers
Animal studies								
1	El-Mahdy et al. [21]	Animal (mice C57BL/6 J)	100	Air	0, 6, or 24 mg/mL	60 weeks	Electronic cigarette can induce cardiovascular disease similar to conventional cigarette smoking. The severity of toxicity increases with exposure duration and nicotine content.	
2	Espinoza-Derout et al. [22]	Animals (ApoE–/– mice)	5/test group	EC without nicotine/saline aerosol	2.40%	12 weeks	Electronic cigarette with nicotine induce an abnormal increase in ROS levels and mitochondrial DNA mutations associated with cardiac dysfunction and atherogenesis. Electronic cigarette without nicotine did not produce significant effect.	
3	Kuntic et al. [23]	Animal (C57BL/6 and NOX2 null mice)	151	Air	12 mg/ml	5 days	Electronic cigarette vapor increases vascular, cerebral, and pulmonary oxidative stress via a NOX-2-dependent mechanism. The adverse effect is more pronounced with nicotine than without nicotine.	
4	Li et al. [24]	Animal (mice)	5-10/test group	Air	2.40%	16 weeks	Electronic cigarette increases the level of mitochondrial DNA damage in blood and expression of TLR9 and induces the development of atherosclerosis.	
5	Olfert et al. [25]	Animal (mice C57BL/6 J)	15	Air	18 mg/mL	32 weeks	Electronic cigarette vapor accelerates arterial stiffness and impairs aortic endothelial function.	
6	Szostak et al. [26]	Animals (ApoE–/– mice)	309 (10-16/test group)	Air	4%	24 weeks	Electronic cigarette vapor shows small or completely absent effects on systolic and diastolic functions of the heart, atherosclerotic progression, altered lipid profiles, and alteration of the heart ventricle and aorta transcriptomes compare to 3R4F conventional cigarette smoke.	

Notes: (∗) The SYRCLE's RoB for animal intervention does not provide overall risk of bias.

**Table 2:** Risk of bias assessment of 3 randomized controlled trial studies using RoB 2.

Criteria	George et al. [11]	Ikonomidis et al. [12]	Ikonomidis et al. [4]
Bias arising from the randomization process	Some concerns	Some concerns	High risk
Bias due to deviations from intended interventions	Some concerns	Some concerns	Low risk
Bias due to missing outcome data	Low risk	Low risk	Low risk
Bias in measurement of the outcome	Low risk	Low risk	Low risk
Bias in selection of the reported result	Low risk	Some concerns	Some concerns
Overall risk of bias	Some concerns	Some concerns	High risk

**Table 3:** Risk of bias (quality) assessment of 7 observational (cross-sectional) studies using NIH Quality Assessment.

Criteria	Bricknell et al. [14]	Fetterman et al. [15]	Oliveri et al. [16]	Podzolkov et al. [17]	Rader et al. [18]	Sahota et al. [19]	Vindhyal et al. [20]
Was the research question or objective in this paper clearly stated?	Yes	Yes	Yes	Yes	No	Yes	Yes
Was the study population clearly specified and defined?	Yes	Yes	Yes	Yes	No	Yes	Yes
Was the participation rate of eligible persons at least 50%?	Yes	Yes	No	Yes	Not reported	Yes	Not reported
Were all the subjects selected or recruited from the same or similar populations (including the same time period)? Were inclusion and exclusion criteria for being in the study prespecified and applied uniformly to all participants?	Yes	Yes	Yes	Yes	Not reported	Yes	Yes
Was a sample size justification, power description, or variance and effect estimates provided?	No	No	Yes	No	No	No	No
For the analyses in this paper, were the exposure(s) of interest measured prior to the outcome(s) being measured?	No	No	No	No	No	No	No
Was the timeframe sufficient so that one could reasonably expect to see an association between exposure and outcome if it existed?	No	No	No	No	No	No	No
For exposures that can vary in amount or level, did the study examine different levels of the exposure as related to the outcome (e.g., categories of exposure, or exposure measured as continuous variable)?	Not applicable	Not applicable	Not applicable	Not applicable	Not applicable	Not applicable	Not applicable
Were the exposure measures (independent variables) clearly defined, valid, reliable, and implemented consistently across all study participants?	No	No	No	No	No	No	No
Was the exposure(s) assessed more than once over time?	No	No	No	No	No	No	No
Were the outcome measures (dependent variables) clearly defined, valid, reliable, and implemented consistently across all study participants?	No	Yes	Yes	Yes	Yes	Yes	No
Were the outcome assessors blinded to the exposure status of participants?	Not applicable	Yes	Yes	Yes	Not reported	Not reported	Not applicable
Was loss to follow-up after baseline 20% or less?	Not applicable	Not applicable	Not applicable	Not applicable	Not applicable	Not applicable	Not applicable
Were key potential confounding variables measured and adjusted statistically for their impact on the relationship between exposure(s) and outcome(s)?	Yes	Yes	Yes	Not applicable	Not applicable	Not applicable	Yes
Overall quality rating	Fair	Good	Good	Fair	Poor	Fair	Poor

**Table 4:** Risk of bias assessment of 6 animal studies using SYRCLE's RoB.

Criteria	El-Mahdy et al. [21]	Espinoza-Derout et al. [22]	Kuntic et al. [23]	Li et al. [24]	Olfert et al. [25]	Szostak et al. [26]
Random group allocation (selection bias)	Unclear	No	No	No	Unclear	Unclear
Groups similar at baseline (selection bias)	Yes	Unclear	Unclear	Unclear	Yes	Yes
Blinded group allocation (selection bias)	No	No	No	No	No	No
Random housing (performance bias)	No	No	No	No	No	No
Blinded intervention (performance bias)	Unclear	Unclear	Unclear	Unclear	Unclear	Unclear
Random outcome assessment (detection bias)	Unclear	Unclear	Unclear	Unclear	Unclear	Unclear
Blinded outcome assessment (detection bias)	No	No	No	No	No	No
Reporting of drop-outs (attrition bias)	Yes	Yes	Yes	Yes	Yes	Yes
Selective outcome reporting	Yes	Yes	Yes	Yes	Yes	Yes
Other biases	Yes	Yes	Yes	Yes	Yes	Unclear

Notes: In the SYRCLE's RoB, ‘Yes' indicates low risk of bias; No indicates high risk of bias; ‘Unclear' indicates unclear risk of bias.
